# Invasive Group B Streptococcal Disease in South Africa: Importance of Surveillance Methodology

**DOI:** 10.1371/journal.pone.0152524

**Published:** 2016-04-07

**Authors:** Vanessa Quan, Jennifer R. Verani, Cheryl Cohen, Anne von Gottberg, Susan Meiring, Clare L. Cutland, Stephanie J. Schrag, Shabir A. Madhi

**Affiliations:** 1 National Institute for Communicable Diseases of the National Health Laboratory Service, Johannesburg, South Africa; 2 Centers for Disease Control and Prevention, Atlanta, Georgia, United States of America; 3 Faculty of Health Sciences, University of the Witwatersrand, Johannesburg, South Africa; 4 Medical Research Council: Respiratory and Meningeal Pathogens Research Unit, Faculty of Health Sciences, University of the Witwatersrand, Johannesburg, South Africa; 5 Department of Science and Technology / National Research Foundation: Vaccine Preventable Diseases, Gauteng, South Africa; Faculdade de Medicina de Lisboa, PORTUGAL

## Abstract

Data on neonatal group B streptococcal (GBS) invasive disease burden are needed to refine prevention policies. Differences in surveillance methods and investigating for cases can lead to varying disease burden estimates. We compared the findings of laboratory-based passive surveillance for GBS disease across South Africa, and for one of the provinces compared this to a real-time, systematic, clinical surveillance in a population-defined region in Johannesburg, Soweto. Passive surveillance identified a total of 799 early-onset disease (EOD, <7 days age) and 818 LOD (late onset disease, 7–89 days age) cases nationwide. The passive surveillance provincial incidence varied for EOD (range 0.00 to 1.23/1000 live births), and was 0.03 to 1.04/1000 live births for LOD. The passive surveillance rates for Soweto, were not significantly different compared to those from the systematic surveillance (EOD 1.23 [95%CI 1.06–1.43] vs. 1.50 [95%CI 1.30–1.71], respectively, rate ratio 0.82 [95%CI 0.67–1.01]; LOD 1.04 [95% CI 0.90–1.23] vs. 1.22 [95%CI 1.05–1.42], rate ratio 0.85 [95% CI 0.68–1.07]). A review of the few cases missed in the passive system in Soweto, suggested that missing key identifiers, such as date of birth, resulted in their omission during the electronic data extraction process. Our analysis suggests that passive surveillance provides a modestly lower estimate of invasive GBS rates compared to real time sentinel-site systematic surveillance, however, this is unlikely to be the reason for the provincial variability in incidence of invasive GBS disease in South Africa. This, possibly reflects that invasive GBS disease goes undiagnosed due to issues related to access to healthcare, poor laboratory capacity and varying diagnostic procedures or empiric antibiotic treatment of neonates with suspected sepsis in the absence of attempting to making a microbiological diagnosis. An efficacious GBS vaccine for pregnant women, when available, could be used as a probe to better quantify the burden of invasive GBS disease in low-middle resourced settings such as ours. From our study passive systems are important to monitor trends over time as long as they are interpreted with caution; active systems give better detailed information and will have greater representivity when expanded to other surveillance sites.

## Introduction

Group B *Streptococcus* (GBS) is a leading cause of neonatal sepsis and meningitis in high-income countries. The epidemiology of invasive GBS disease in low- and middle-income countries is less well characterized [1]. Several studies have demonstrated the importance of GBS as a neonatal pathogen in sub-Saharan Africa, with incidence of invasive early-onset (EOD, between 0–6 days of life) and late-onset (LOD, from 7 to 89 days of life) disease of 0.92 to 2.09 and 0.56 to 1.0 per 1,000 live births, respectively [2–4]. Studies from some regions, including West Africa and South Asia, have, however, reported much lower incidence of invasive GBS disease [1,5] despite evidence that maternal colonization with GBS does not vary significantly by geographic region [6].

Accurate data on disease burden are important for prioritization of prevention strategies such as the development of a maternal GBS vaccine [2,7]. Suboptimal surveillance can produce misleading results that can negatively impact policies. To examine the impact of surveillance methods on disease burden estimates, we compared the findings of laboratory-based passive surveillance for GBS disease across South Africa with that of real-time, systematic clinical and laboratory-based surveillance at a major South African secondary-tertiary care hospital serving a defined population [8].

## Methods

The National Health Laboratory Service (NHLS) provides diagnostic laboratory services for all public-sector hospitals, which serve approximately 80% of the South African population. Test results are stored in an electronic repository (Corporate Data Warehouse [CDW]). Data from NHLS laboratories throughout the country are automatically uploaded onto the CDW. Data not captured at the source laboratory (e.g., date of birth, gender) are missing in CDW. While the ward listed in CDW could provide some indication of the general age group (e.g. neonatal or paediatric), this information was not specific enough to classify cases as either EOD or LOD, and thus was not utilized in the analysis.

We searched CDW for all reports of GBS isolated from a normally-sterile specimen (e.g., cerebrospinal fluid, blood) or detected by latex agglutination plus Gram stain suggestive of GBS from January 2004 through December 2008. For that time period, CDW included data for 8 of 9 South African provinces (all except KwaZulu-Natal [KZN]). Persons with multiple positive specimens from a single episode of illness (defined as specimens collected within 21 days of the first positive GBS result) were counted as one case. Duplicates were removed using names, laboratory numbers as well as dates of birth for both data bases. Identifiers were included prior to CDW analysis but stripped out prior to the CHBAH analysis. The date used to calculate onset in CDW was based on the lab processing date, therefore the exact onset of illness could not be known. This may also have affected the numbers of EOD and LOD.

Numbers of live births [9] excluding live births in KZN (because KZN was not on a laboratory information system and hence there were no data in CDW) were used to estimate rates of early- and late-onset disease, defined as cases occurring among infants aged 0 to 6 and 7 to 89 days, respectively. Home births are included in the national data. In 2010, approximately 87.3% of births nationally occurred in health care facilities [10]. These data represented passive GBS surveillance.

**Fig 1:**
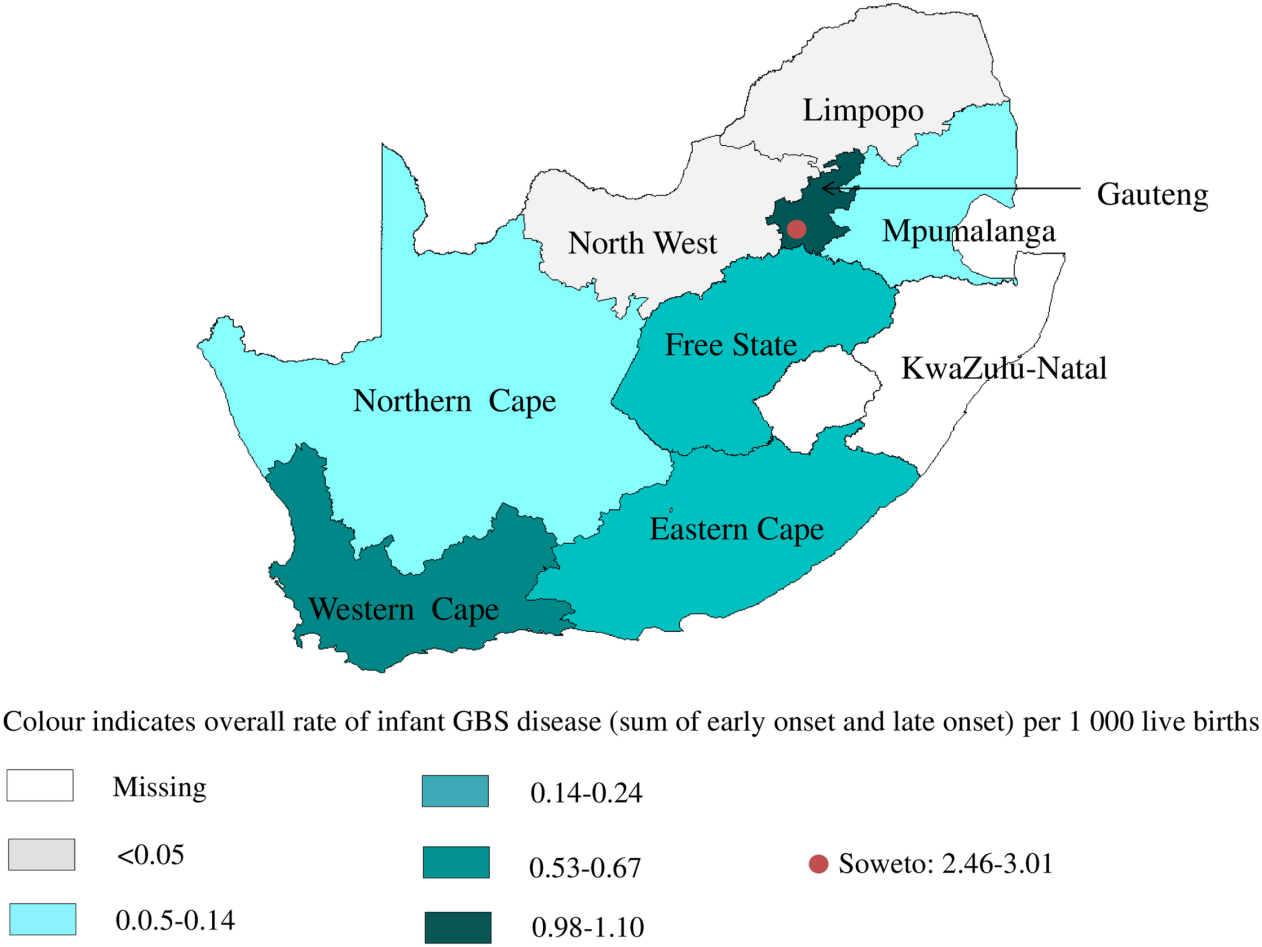
Chloropleth map of South Africa demarcating provinces (and Soweto) and their respective rates of infant GBS disease, 2004–2008.

Chris Hani Baragwanath Academic Hospital (CHBAH) is a 3000-bed academic hospital affiliated with the University of the Witwatersrand, in Soweto, Johannesburg (Fig 1). It is the only public-hospital in Soweto with neonatal care facilities and approximately 90% of all hospitalizations from the community occur at this hospital. The annual birth cohort of Soweto is approximately 30 000. Ninety five per cent of deliveries in Soweto occur in health facilities, the majority (approximately 23 500) occur at CHBAH, and the remainder at seven surrounding midwife operated units (MOUs). The nursing staff at the MOU has a low threshold for referring any women experiencing difficulty with progress in labour and all newborns with respiratory distress or other illness directly to CHBAH for further management. [8,11] Furthermore, during the time period of this study, CHBAH was the only public secondary/tertiary hospital in the Soweto area and the majority of infants, few (<10%) of whom are on private medical insurance would have sought medical care directly at the hospital or would have been referred from the primary health clinics if requiring hospitalization. Although CHBAH is also a referral hospital for the region, neonatal sepsis is not considered specialised care and is generally managed at secondary hospitals in each province. Health care in South Africa, particularly for acute conditions such as labour and neonatal sepsis, is generally sought at the hospital serving specific catchment areas where the individual resides. Hence, the majority of EOD and LOD cases at CHBAH are likely to have been in infants resident in Soweto itself.

The standard of care for prevention of invasive GBS disease at CHBAH, only involves recommending IAP (intravenous ampicillin and oral metronidazole) for women in labour with suspected chorioamnionitis and prolonged rupture of membranes. During this study period, 10.5% of women received IAP during labour [8]. IAP practices vary in the country and it is difficult to assess what proportion of women in labour, at institutions with formal guidelines, actually receive IAP.

Real-time, systematic clinical and laboratory-based surveillance for invasive GBS disease has been on-going since 2004 at CHBAH. Here, attending clinicians have a low threshold for undertaking blood and other sterile site cultures from newborns with suspected sepsis. All positive isolates were checked against the laboratory log daily to identify the invasive GBS cases. The new cases were tracked and additional confirmatory data (including date of birth and gender) were added to the CHBAH database, but not to the CDW if missing. Twice a year, all invasive GBS cases were drawn down from the electronic database at CHBAH laboratory and these cases were cross-matched with the cases identified through the systematic surveillance platform (personal communication CL Cutland). The microbiology laboratory at CHBAH is accredited by the South African National Accreditation System (SANAS) [12].

We considered published data from CHBAH to be the referent standard for GBS EOD and LOD incidence in South Africa.

We used previously-unpublished data from January 2004 through December 2008, extracted from CDW and published data from CHBAH for that time period. In addition we compared the cases identified through CDW for CHBAH to the systematic surveillance identified cases at CHBAH to evaluate the robustness of CDW as a passive surveillance tool.

We calculated incidences of EOD and LOD using CDW data for (1) the CHBAH catchment area, (2) Gauteng province (in which CHBAH is located), and (3) all of South Africa (excluding KZN); we compared CDW CHBAH to CHBAH systematic surveillance incidence using a rate ratio. Denominators for provinces were taken from census data. Confidence intervals were calculated using the Poisson distribution for incidence rates (IR) and log binomial model for incidence rate ratios (IRR).

Although we used only isolate data from patients, we received ethics approval from University of the Witwatersrand Human Research Ethics Committee (M120368). Since it was secondary data analyses, the Ethics committee waived the need for informed consent. All patient records/information was anonymised and de-identified prior to CHBAH analysis. CDW was analysed with identifiers.

## Results

From January 2004 through December 2008, 2,468 cases of invasive GBS were identified in CDW, including 2,197 (89.0%) with available birth date. Of those, 1,617 (73.6%) occurred among neonates aged ≤90 days, including 799 (49.4%) early- and 818 (50.6%) late-onset cases. Among EOD cases in CDW, the median age of case detection was two days; 74 (9.3%) manifested on the day they were born, 301 (37.7%) on day 1 of life and 176 (22%) on day 2 of life; this was based on the laboratory processed date rather than the date of onset of illness and skewed the data especially in the first 48 hours of life. Gender was known in 2144 cases (87%) of whom 1079 (50%) were male; there was no marked difference in gender distribution across provinces. Among neonates aged ≤90 days with known gender, 52% (1428) were male.

The observed case numbers and incidence (per 1 000 live births) using CDW data for the CHBAH catchment area were approximately 18% lower than the real-time systematic clinical-based surveillance at CHBAH incidences for both EOD (1.23 [95%CI 1.06–1.43] vs. 1.50 [95%CI 1.3–1.71]; rate ratios 0.82 [95%CI 0.67–1.01]) and LOD (1.04 [95%CI 0.90–1.23] vs. 1.22 [95%CI 1.05–1.42]; rate ratio 0.85 [95%CI 0.68–1.07]), although the confidence levels for the two surveillance systems overlapped; Table 1.

**Table 1:** Estimates of incidence of early- and late-onset GBS disease and rate ratios and infant disease comparing rates using provincial and overall CDW reports to real-time clinical surveillance at CHBH, 2004–2008.

Data source	Observed values			Infant disease	Early-onset GBS disease		Late-onset GBS disease	
Item	Numerator	Numerator	Denominator‡	Incidence per 1,000 live births (95% CI)	Incidence per 1,000 live births (95% CI)	Rate ratio[i] (95% CI)	Incidence per 1,000 live births (95% CI)	Rate ratio (95% CI)
	Early-onset	Late-onset						
Cases detected through systematic clinical surveillance at CHBAH, Soweto [8]	214	175	142,883	2.72	1.5 (1.30–1.71)	1	1.22 (1.05–1.42)	1
Cases reported in CDW from CHBAH	176	150	142,883	2.28	1.23 (1.06–1.43)	0.82 (0.67–1.01)	1.04 (0.90–1.23)	0.85 (0.68–1.07)
Cases reported in CDW from Gauteng province	535	495	992,288	1.04	0.54 (0.49–0.59)	0.36 (0.31–0.42)	0.49 (0.46–0.54)	0.40 (0.34–0.49)
Cases reported in CDW in all provinces[ii]	799	818	4,261,138	0.38	0.19 (0.17–0.20)	0.13 (0.11–0.15)	0.19 (0.18–0.21)	0.16 (0.13–0.19)
Cases reported in CDW in Eastern Cape Province	67	65	800,088	0.17	0.08 (0.06–0.10)	0.05 (0.04–0.07)	0.08 (0.06–0.10)	0.07 (0.05–0.08)
Cases reported in CDW in Free State Province	29	27	301,818	0.19	0.10 (0.06–0.13)	0.07 (0.05–0.08)	0.09 (0.06–0.13)	0.07 (0.05–0.10)
Cases reported in CDW in Limpopo Province	3	23	737,489	0.04	0	0	0.03 (0.02–0.05)	0.02 (0.01–0.03)
Cases reported in CDW in Mpumalanga Province	25	27	420,242	0.12	0.06 (0.04–0.09)	0.04 (0.03–0.06)	0.06 (0.04–0.09)	0.05 (0.03–0.7)
Cases reported in CDW in North West Province	1	11	407,096	0.03	0	0	0.03 (0.01–0.05)	0.02 (0.01–0.03)
Cases reported in CDW in Northern Cape Province	5	6	103,153	0.11	0.05 (0.02–0.11)	0.03 (0.02–0.06)	0.06 (0.02–0.13)	0.05 (0.03–0.07)
Cases reported in CDW in Western Cape Province	134	164	498,965	0.6	0.27 (0.23–0.32)	0.18 (0.17–0.24)	0.33 (0.28–0.38)	0.27 (0.20–0.30)

^i^ Compared with data from real-time clinical GBS surveillance at CHBAH from 2004–2008 [8]

^ii^ Excluding Kwa-Zulu Natal

^‡^ Live births excluding Kwa-Zulu Natal

Denominators used for CHBAH rates are Soweto live births; for provinces are provincial live births

When the CHBAH active surveillance and the CDW database were compared, 355 (91%) of the active surveillance cases were matched to the CDW database. Using the dates of birth from the active-surveillance database, an additional 34 CDW cases (33 EOD and 1 LOD) were identified.

Rates (per 1000 live births) for EOD and LOD varied considerably by province (Table 1), with the highest in Gauteng (0.54 [95%CI 0.49–0.59] and 0.49 [95%CI 0.46–0.54], respectively), and the lowest in Limpopo (0 and 0.03 [95%CI 0.02–0.05]) and North West province (0 and 0.03 [95%CI 0.01–0.05]). The Western Cape rates were 0.27 [95%CI 0.23–0.32] and 0.33 [95%CI 0.28–0.8] for early- and late-onset disease, respectively. The infant disease rates are shown in Table 1 and Fig 1; Soweto rates being much higher than elsewhere.

## Discussion

In this assessment of the burden of invasive GBS disease in South Africa, we found marked differences across provinces, with implausibly low estimates of disease from many provinces. Passive laboratory-based surveillance (CDW) provided reasonable estimates of the burden of invasive GBS disease when restricted to an area in which clinical and laboratory practices are conducive to identifying GBS disease e.g., CHBAH [8], This, however, would have under-estimated the incidence of EOD and LOD by 18% and 15%, respectively. This discrepancy could be due to by missing data in the CDW; for example, a missing date of birth would be tracked down in the active surveillance, but the case would have been omitted when interrogating CDW data alone in the presence of age as a filtering search criteria. There were an additional 34 cases that the active surveillance system picked up but were not on the initial CDW database; especially in 2004. This may have been due to a change-over in the laboratory information systems at the time or that there was a dedicated person doing the same drawdown six monthly at CHBAH whereas the CDW drawdown was done as a once off many years later.

Considering the public health care structure in South Africa, the high incidence of invasive GBS disease reported for Soweto is unlikely to be driven by large numbers of neonates resident outside of Soweto seeking care at CHBAH. This is corroborated in that the overall incidence (per 1000 live births) of invasive GBS disease reported for Soweto using the sentinel site systematic surveillance (2.72) [8], was similar to that observed in a cohort study of 8000 pregnant women delivering at CHBAH (2.0) [13]. Rather, this high incidence is likely due to the majority of births in Soweto occurring at a secondary-tertiary levels hospital, with a low threshold of referring ill neonates from surrounding primary health care clinics. This is further coupled with a low threshold for undertaking microbiological tests, including blood and cerebrospinal fluid culture, in neonates with suspected sepsis and prior to initiating antibiotic treatment. Furthermore, this is supported by an accredited laboratory which uses state-of the art automated blood culture methods.

Passive surveillance data from other provinces yielded markedly lower incidence estimates, with remarkably few EOD recorded in some provinces. Although it is possible that some true geographic variability in GBS burden exists within South Africa, e.g., in the Western Cape where we assume good blood culturing practices and low disease rates [4], particularly since HIV exposure has been shown to be a risk factor for GBS disease and the prevalence of HIV among pregnant women varies across provinces [8]. However, the magnitude of the differences we observed suggests that in many regions of South Africa, invasive GBS disease goes largely undiagnosed possibly due to blood culturing practices. This is supported in that the prevalence of vaginal GBS colonization, which is the key risk factor for EOD, has been reported to be similar in different provinces in South Africa [14]. Consequently, when considering that approximately 50% of newborns to GBS colonized women are colonised at birth, of whom 1–3% develop invasive EOD in the absence of IAP [15], it would have been expected that the incidence of EOD would have been similar between the provinces, in the absence of screening for GBS during pregnancy and only a risk-based IAP strategy. These estimates of 1–3% of colonized new-borns developing EOD were also corroborated in a study from Soweto, in which of the 57% of newborns born to colonised women were colonised themselves at birth, of whom 2% developed EOD [14].

It might be that differences in practice for investigating neonates with suspected sepsis is a critical determinant to the variability in EOD incidence in South Africa. We did a neonatal sepsis survey in 2012 where 17 paediatricians from secondary/tertiary centres throughout the country were asked about specimen-taking practices in sick children (unpublished data). Fourteen sites (82%) said they did diagnostic evaluations (including blood culture but not routine CSF culture) on obviously septic neonates >75% of the time. Three sites said they did not do blood cultures on all septic neonates and these are the respective secondary hospitals in the 3 provinces that had the fewest GBS cases (LP, NW and NC).

The timing of early-onset cases in this study also suggests a failure to detect cases occurring within the first 24 hours of life. Among all newborns with early-onset disease within CDW, only 9.3% had specimens collected on the same date they were born. In contrast, at CHBAH, 64.5% of early-onset cases were detected within the first 24 hours [8]. The data from CHBAH are consistent with data from the Unites States of America, where before widespread GBS prevention efforts approximately 80% of early-onset cases occurred within the first 24 hours of life, with the majority of those presenting within hours of being born [15]. The relative lack of cases detected within the first 24 hours of life in the CDW data suggests that in much of the country, GBS presenting soon after birth may be frequently missed and babies born with respiratory distress should be investigated more aggressively.

Several factors may contribute to the under-ascertainment of GBS cases [5]. Babies born at home, limited access to health care particularly for newborns, or empiric treatment at primary level health facilities may affect the number of cases detected. In Soweto, the primary area served by CHBAH, medical care is relatively accessible [16] as it is in the WC tertiary centres [4], although in the WC the majority of births occur at mid-wife operated units and ill neonates are empirically initiated on antibiotic prior to being transferred to a secondary or tertiary care hospital, where blood and CSF cultures might be obtained. Also, in other areas of South Africa there may be more barriers to care for ill neonates and a higher proportion of births born outside of health facilities. Variability in laboratory capacity is likely also an important factor in the South African context, particularly for babies born at primary health care facilities where blood culture facilities are generally unavailable, inadequate specimen collection, laboratory supplies and suboptimal laboratory methods also play a part especially in district hospitals.

Early-onset GBS disease can be prevented through intravenous intrapartum antibiotics for GBS-colonized mothers [17]. While this strategy is not feasible in most resource-poor settings because of the cost and complexity of implementation, a risk-based approach using intrapartum risk factors (rather than pre-natal screening) to guide antibiotic prophylaxis is currently used in several hospitals across South Africa, including CHBAH, with limited success [16]. Risk-based IAP could potentially have impacted observed rates of disease; however the coverage of this prevention intervention at CHBAH is likely similar or greater than coverage nationwide, yet CHBAH had the highest measured rates. Therefore heightened use of IAP elsewhere in SA, is unlikely to have contributed to the variability in GBS disease incidence observed.

This study has several limitations. Only one site with systematic surveillance was available to compare against a passive surveillance system using CDW. No formal surveillance evaluation was done to look at system attributes of simplicity, flexibility, data quality, acceptability, sensitivity, predictive value positive, representativeness, timeliness and stability. There were no published data on specimen taking practices countrywide other than the short survey we did (unpublished data).

Limitations to the passive CDW surveillance system are that it is laboratory-based and only includes patients who sought care at public hospitals and where blood cultures were done and a sufficient laboratory capacity was established. It is therefore an underestimate of the true national burden [18]. In addition, many patients will seek care at the local clinic before referral to the second/tertiary level centre; here antibiotics may be given before referral hence even if they have blood cultures taken these will be negative. Problems with data quality in CDW affected our findings. For example, differing information on laboratory number and missing date of birth in CDW resulted in cases being missed, since without date of birth cases could not be classified as early- or late-onset. CDW had 11% of birth dates and 13% of gender missing. Provincial population live birth data may not be as accurate as the Soweto population live birth data used as the denominator for CHBAH, resulting in over- or underestimates of incidence, depending upon the direction of the error.

The variability in rates highlights the need to interpret GBS data obtained through passive surveillance with caution. Passive surveillance systems may have the benefit of being feasible and low cost, which are important considerations in resource-poor areas. They also may be used to monitor changes over time if the methods are stable. It cannot, however, address factors such as threshold for investigating and is subject to temporal changes which could influence trends over time. Undertaking sentinel surveillance studies, with more patient details, to measure the true burden of disease is useful as has been done in Soweto. Expanding this to other surveillance sites would improve the representivity of data. Where suboptimal clinical and laboratory practices are in place, it is easy to conclude that GBS disease is not an important public health problem. Policy decisions and efforts to develop new GBS prevention strategies should be guided only by high-quality surveillance data that are likely to yield a more accurate estimation of true disease burden.

Vaccine probe studies for pneumococcus and *Haemophilus influenzae* type b invasive disease, illness which also suffer from under detection because of diagnostic limitations, have been useful in defining more accurate estimates of the burden of disease caused by these organisms [19]. These studies look at the difference between the burden of disease in vaccinated and unvaccinated persons. This difference can then be attributed to that vaccine-specific pathogen. Maternal GBS vaccines are being pursued as a prevention measure for neonatal disease [20]. Once a safe and efficacious vaccine is available, a probe study could be useful in characterising the burden of GBS disease, particularly in resource-poor settings. Until that time, data on neonatal GBS disease from surveillance systems that are not optimised should be interpreted with caution.

## Supporting Information

S1 Table The minimal data set is attached as supporting information. (XLSX)

## References

[1] Dagnew AF, Cunnington MC, Dube Q, Edwards MS, French N, Heyderman RS, et al (2012) Variation in reported neonatal group B streptococcal disease incidence in developing countries. Clin Infect Dis 55: 91–102. 10.1093/cid/cis395 22523262

[2] Madhi SA, Radebe K, Crewe-Brown H, Frasch CE, Arakere G, Mokochane M, et al (2003) High burden of invasive Streptococcus agalactiae disease in South African infants. Ann Trop Paediatr 23: 15–23. 12648320 10.1179/000349803125002814

[3] Gray KJ, Bennett SL, French N, Phiri AJ, Graham SM (2007) Invasive group B streptococcal infection in infants, Malawi. Emerg Infect Dis 13: 223–229. 17479883 10.3201/eid1302.060680 PMC2725867

[4] Frigati L, van der Merwe JL, Harvey J, Rabie H, Theron G, Cotton MF(2014) A retrospective review of group B streptococcal infection in teh Metro East area of the Western Cape province: 2010 to 2011. South Afr J Infect Dis 29: 33–36.

[5] Edmond KM, Kortsalioudaki C, Scott S, Schrag SJ, Zaidi AK, Cousens S, et al (2012) Group B streptococcal disease in infants aged younger than 3 months: systematic review and meta-analysis. Lancet 379: 547–556. 10.1016/S0140-6736(11)61651-6 22226047

[6] Kwatra G CM, Valencia C, Adrian PV, Ip M, Klugman K, Madhi SA, Tam WM. Slobod K. (2013) Maternal colonization with Group B streptococcus: Do rates vary across regions? 8th World Congress of World Society for Pediatric Infectious Diseases. Cape Town, South Africa.

[7] Schrag SJ, Global Group BSVWG (2011) Group B streptococcal vaccine for resource-poor countries. Lancet 378: 11–12. 10.1016/S0140-6736(10)61932-0 21377722

[8] Cutland CL S S, Thigpen MC, Velaphi SC, Wadula J, Adrian PV, Kuwanda L, Groome MJ, Buchmann E, Madhi SA. (2015) Increased Risk for Group B Streptococcus Sepsis in Young Infants Exposed to HIV, Soweto, South Africa, 2004–2008. Emerg Infect Dis 21: 638–645. 10.3201/eid2104.141562 25812061 PMC4378461

[9] Actuarial Society of South Africa (2011) ASSA 2008 model 2011. [cited 2014 14 June]. Available from: http://aids.actuarialsociety.org.za/

[10] Goals. NCCftMD (2014) Millenium development goals, country report 2013 STATSSA2014. [cited 2015 9 November]. Available from http://www.statssa.gov/za/wp-content/uploads/2014/02/MDGR_Report_2013_Final.pdf

[11] Cutland CL, Madhi SA, Zell ER, Kuwanda L, Laque M, Groome M, et al (2009) Chlorhexidine maternal-vaginal and neonate body wipes in sepsis and vertical transmission of pathogenic bacteria in South Africa: a randomised, controlled trial. Lancet 374: 1909–1916. 10.1016/S0140-6736(09)61339-8 19846212

[12] SANAS (2015). Available from: home.sanas.co.za

[13] Cutland CL, Schrag SJ, Zell ER, Kuwanda L, Buchmann E, Velaphi S, et al (2012) Maternal HIV infection and vertical transmission of pathogenic bacteria. Pediatrics 130: e581–590. 22869824 10.1542/peds.2011-1548

[14] Madhi SA, Dangor Z, Heath PT, Schrag S, Izu A, Sobanjo-Ter Meulen A, et al (2013) Considerations for a phase-III trial to evaluate a group B Streptococcus polysaccharide-protein conjugate vaccine in pregnant women for the prevention of early- and late-onset invasive disease in young-infants. Vaccine 31 Suppl 4: D52–57. 10.1016/j.vaccine.2013.02.029 23973347

[15] Schuchat A (1998) Epidemiology of group B streptococcal disease in the United States: shifting paradigms. Clin Microbiol Rev 11: 497–513. 9665980 10.1128/cmr.11.3.497 PMC88893

[16] Schrag SJ, Cutland CL, Zell ER, Kuwanda L, Buchmann EJ, Velaphi S, et al (2012) Risk factors for neonatal sepsis and perinatal death among infants enrolled in the prevention of perinatal sepsis trial, Soweto, South Africa. Pediatr Infect Dis J 31: 821–826. 10.1097/INF.0b013e31825c4b5a 22565291

[17] Verani JR, McGee L, Schrag SJ, Division of Bacterial Diseases NCfI, Respiratory Diseases CfDC, et al (2010) Prevention of perinatal group B streptococcal disease—revised guidelines from CDC, 2010. MMWR Recomm Rep 59: 1–36. 21088663

[18] von Gottberg A, de Gouveia L, Tempia S, Quan V, Meiring S, von Mollendorf C, et al (2014) Effects of vaccination on invasive pneumococcal disease in South Africa. N Engl J Med 371: 1889–1899. 10.1056/NEJMoa1401914 25386897

[19] Feiken D, Scott JAG, Gessner BD (2014) Use of vaccines as probes to define disease burden. The Lancet 383: 1762–1770. 10.1016/S0140-6736(13)61682-7 PMC4682543 24553294

[20] Heyderman RS, Madhi S. A., French N., Cutland C., Ngwira B., Kayambo D., Mboizi R., Koen A., Jose L., Olugbosi M., Wittke F., Slobod K., Dull P. M. A Phase II Open-Label, Multi-Center Study of a Group B Streptococcus Vaccine in HIV-infected and HIV-uninfected Pregnant Women in Africa. Lancet Infect Dis 2015 (in press). 10.1016/S1473-3099(15)00484-3 PMC4835545 26869376

